# Evaluation of Intravenous Immunoglobulin Administration for Hyperbilirubinemia in Newborn Infants with Hemolytic Disease

**DOI:** 10.3390/children10030496

**Published:** 2023-03-02

**Authors:** Daniel R. Mohan, Hannah Lu, Jacquelyn McClary, Jaime Marasch, Mary L. Nock, Rita M. Ryan

**Affiliations:** 1 Department of Pediatrics (Neonatology), Rainbow Babies and Children’s Hospital, Cleveland, OH 44106, USA; 2 Department of Pharmacy, Rainbow Babies and Children’s Hospital, Cleveland, OH 44106, USA; 3 Department of Pediatrics, Case Western Reserve University, Cleveland, OH 44106, USA

**Keywords:** bilirubin, Coombs test, hyperbilirubinemia, intravenous immunoglobulin, neonatal intensive care unit, phototherapy, retrospective studies

## Abstract

The primary objective of this research was to evaluate the use of intravenous immunoglobulin (IVIG) in infants with hemolytic disease, to assess compliance with the American Academy of Pediatrics (AAP) guideline recommendations, and to review the data on which the guidelines were based. This retrospective study evaluated all infants in the NICU (neonatal intensive care unit) who received IVIG between January 2018 and December 2020 (*n* = 71). Total serum bilirubin (TSB) levels surrounding the time of IVIG administration, rate of rise of bilirubin, and direct antiglobulin test (DAT) status were evaluated to determine the appropriateness of IVIG use based on the 2004 AAP recommendations that was current at the time of the study. Fifty-nine infants received IVIG for hyperbilirubinemia. Of them, 80% had an ABO mismatch, 19% had Rh mismatch, and 71% were DAT-positive. Phototherapy was started at an average of 7 h of age, and the first IVIG dose was administered at an average of 13 h of life; nearly 25% received a second IVIG dose. One infant (1.6%) met all three AAP guideline criteria of being DAT-positive, bilirubin within 3 of exchange level, and rising bilirubin despite intensive phototherapy. Twenty-five (42%) babies were DAT positive and met one of the other two criteria. Only 12% (*n* = 7) had a bilirubin within 3 of exchange level. Most infants who received IVIG for hyperbilirubinemia did not meet the AAP criteria, prompting us to develop an institution-specific IVIG clinical practice guideline. The 2022 AAP guideline was published after our study was completed, but it confirmed our belief that IVIG usage should be more restricted and the criteria more explicit.

## 1. Introduction

Isoimmune hemolytic disease is a significant cause of hyperbilirubinemia and hospitalization in newborns. Intensive phototherapy and, in severe cases, double-volume exchange transfusion (DVET), are used to treat acute bilirubin encephalopathy and reduce kernicterus. Intravenous immunoglobulin (IVIG) is a treatment option for isoimmune hemolytic hyperbilirubinemia with the aim of reducing exchange transfusions [1]. The proposed mechanism for IVIG in treating hyperbilirubinemia involves nonspecific blockade of Fc receptors on reticulo-endothelial cells, leading to a decrease in red blood cell destruction and decreasing their contribution to bilirubin production [2]. However, despite the use of prophylactic IVIG in isoimmune hemolytic disease, studies have shown limited and conflicting data on the efficacy in decreasing the need for exchange transfusions [1]. In addition, IVIG use has been associated with a range of adverse effects, incurs significant cost per dose, and exposes the neonate to a large number of donors [3,4,5,6,7,8]. The previous American Academy of Pediatrics (AAP) hyperbilirubinemia guideline (2004) [9], which was in place during the time these patients were in our care, states,

*“In isoimmune hemolytic disease, administration of intravenous γ-globulin (0.5–1 g/kg over 2 h) is recommended if the total serum bilirubin (TSB) is rising despite intensive phototherapy, or the TSB level is within 2 to 3 mg/dL (34–51 mol/L) of the exchange level. If necessary, this dose can be repeated in 12 h.”*

While the exchange level was clearly described in the guideline via a nomogram, there was not a clear definition of how to diagnose “isoimmune hemolytic disease.” The randomized trials demonstrating efficacy for IVIG were all in infants with a positive direct antiglobulin test (DAT) and either Rh or ABO hemolytic disease of the newborn (HDN) (Table 1). The duration of “intensive phototherapy” (defined as an irradiance of 30 µW/cm/nm or higher in the 430–490 nm wavelength, delivered to as much of the infant’s skin surface area as possible) prior to IVIG administration was also not specified in the AAP guideline [9]. In our neonatal intensive care unit (NICU), irradiance is checked and monitored frequently. Since it was not defined in the AAP guidelines specifically, we used four h of phototherapy as the length of time to analyze whether the bilirubin is “rising despite intensive phototherapy”.

Given the concern of potential adverse events, significant cost, and donor exposure of IVIG therapy, a single-center retrospective study was performed to see how our clinicians’ administration of IVIG compares to the 2004 guidelines recommended by the AAP, with the plan to develop an institution-specific guideline if needed. The primary objective was to evaluate the use of IVIG in newborn infants with hemolytic disease. Secondary objectives included the evaluation of the adverse effects of IVIG.

We undertook this investigation to evaluate our clinical practice use of IVIG for immune-mediated hemolytic hyperbilirubinemia and found that there was room for improvement. We offer these data to heighten awareness regarding the general knowledge of using IVIG for HDN and interpretation of the 2004 AAP guidelines. During submission, the new AAP 2022 guidelines were published, and we have incorporated some of the changes related to IVIG usage in this report.

## 2. Materials and Methods

### 2.1. Study Design and Participants

This was a single-center retrospective study conducted at UH Rainbow Babies and Children’s Hospital and Case Western Reserve University in an 82-bed level IV NICU. Patients who received at least one dose of IVIG between January 2018 and December 2020 in the NICU were identified from the electronic medical record and were excluded from the study if they received IVIG for any indication other than hyperbilirubinemia. The study was approved by the University Hospitals Institutional Review Board.

### 2.2. Data Collection

Demographic information collected included gestational age, sex, birth weight, and ethnicity. To determine the presence of Rh or ABO mismatch, the following information was collected: blood type for the mother and baby, the mother’s antibody screen, and the baby’s DAT results. We collected the number of IVIG doses and the hour of life at which IVIG was ordered and administered in relation to the initiation and duration of phototherapy. To calculate the rate of rise of TSB, we collected the two TSB levels (if available) prior to when IVIG was ordered. TSB levels were entered into a hyperbilirubinemia management tool [10] to identify if the last TSB level prior to the IVIG order was within 3 mg/dL of the exchange transfusion level. The number of exchange transfusions received, if any, was also collected.

### 2.3. Adverse Events

Safety outcome data for IVIG included necrotizing enterocolitis, thrombosis, anaphylaxis, and hypoglycemia during the IVIG infusion [3,4,5,6,7]. Hypoglycemia was defined as blood glucose less than 50 mg/dL.

### 2.4. Statistical Analysis

Descriptive statistics were used; categorical data are presented as frequency and percentages, while continuous data are described as mean ± standard deviation.

This study was a “medication usage” analysis: a descriptive study to examine the use of IVIG in our NICU and our adherence to the AAP 2004 guidelines. Thus, we did not perform a sample size calculation for this study.

## 3. Results

Seventy-three patients who had IVIG ordered between January 2018 and December 2020 were reviewed for inclusion and exclusion criteria. In the final detailed analysis, which included 59 patients who received IVIG for hyperbilirubinemia, the mean gestational age was 39 weeks (Table 2). Rh and ABO “mismatch” were identified in 18.6% (*n* = 11) and 79.7% (*n* = 47) of mother–baby dyads, respectively; 42/59 (71%) were DAT-positive.

The majority of patients received only one dose of IVIG, and essentially all received a dose of 1 g/kg (Table 3). The median hour of age for doses 1, 2, and 3 was ~13, 40, and 80 h, respectively. No patients experienced necrotizing enterocolitis (NEC), thrombosis, or anaphylaxis. Two patients had documented hypoglycemia during administration of IVIG, though 12 of the 59 patients had no documented blood sugar measurements during the infusion. In one of the two infants with hypoglycemia, a second intravenous (IV) line for dextrose-containing fluids had not been placed. When an infant cannot have a second IV line placed and is not feeding enterally (some infants are made NPO when a DVET is a possibility), the infant will go for several hours with no IV glucose while the IVIG infusion is running. Of the 59 patients who received IVIG, 3.4% (*n* = 2) received an exchange transfusion. Two of the patients who were DAT positive were not an Rh or ABO mismatch; one had anti-c (and eventually got DVET) and one had anti-E.

**Figure 1:**
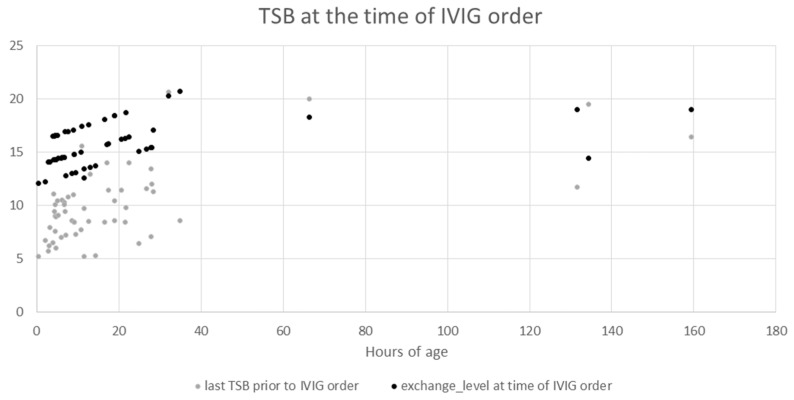
TSB at the time of IVIG order. The y-axis denotes the serum bilirubin in mg/dL with the grey dots indicating the last serum bilirubin prior to IVIG being ordered and the black dots indicating the exchange level at the same time. The x-axis denotes the hour of age the level was obtained.

**Figure 2:**
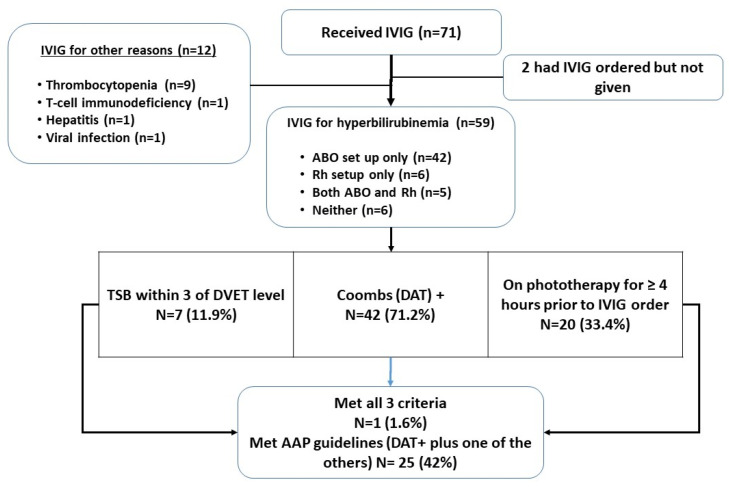
Patient flow diagram and assessment of meeting AAP Guideline criteria for IVIG treatment (abbreviations: DVET, double-volume exchange transfusion; DAT, direct antiglobulin test).

The relationship of bilirubin levels and when IVIG was ordered was explored in more detail (Table 4, and Figure 1). There were four patients with no TSBs in our system; presumably, there were bilirubin levels obtained from an outside office or hospital. We were unable to obtain bilirubin levels drawn at other institutions because our IRB approval for this retrospective study only covered our own data system. The mean TSB prior to the first IVIG order for patients who had one or at least two levels obtained was 9.0 and 10.7, respectively. The mean rate of rise of TSB for those with at least two levels was 0.43, while the median was 0.21. The mean and median value of the last TSB prior to the first IVIG order was 10 and 9.4 (Table 4). By examining the individual TSB and exchange level at the time of each IVIG order, we found that patients with IVIG orders placed at a later hour of life are more often within 3 of exchange level than when compared to those with IVIG doses ordered in the first 40 h of life (Figure 2).

In examining adherence to the 2004 AAP guidelines for the 59 patients included in the detailed analysis, 71% were DAT-positive, which we used as a minimum criterion for “isoimmune hemolytic disease.” Only 11.9% (*n* = 7) of babies had a TSB within 3 mg/dL of the exchange level as stated in the AAP guideline at the time of the first dose of IVIG (Figure 2). Only 20 babies (33.4%) had at least four h of phototherapy prior to IVIG being ordered. For study purposes, we considered having met the exact AAP criteria as being DAT-positive and either within 3 of DVET level or showing a rising bilirubin despite four h of phototherapy. The criteria were met by 25 patients (42%), with only one patient (1.6%) meeting all three criteria (Figure 2).

## 4. Discussion

The most recent Cochrane Review in 2018 showed a statistically significant reduction in the need for DVET in neonates with isoimmune hemolytic disease who were treated with prophylactic IVIG [1]. However, it was noted that the two highest quality studies, which were blinded by using a placebo, failed to show a statistically significant reduction in DVET in neonates treated with IVIG versus intensive phototherapy alone, leading the authors to recommend not using IVIG without additional studies. In addition, we noted that the literature reports that IVIG is associated with a range of adverse effects including anaphylaxis, NEC, and hemolysis [3,4,5,6,7]. For a 3 kg patient, a single dose of IVIG incurs a hospital cost of $684, a patient charge of $3375 (our institution-specific cost data), and the potential for significant donor exposure (1000–15,000 per dose depending on the preparation) [8]. The donor exposure is especially surprising. Based on these costs and possible adverse effects, we sought to determine if our usage of IVIG was consistent with current recommendations and evidence-based studies. We found that we were not consistently using IVIG for immune-mediated hyperbilirubinemia in accordance with the 2004 AAP recommendations and prior evidence-based studies. Particularly concerning was our use of IVIG when the TSB was not within 3 mg/dL of the exchange transfusion level.

Our extensive use of IVIG was anecdotally related to the primary goal of avoiding an exchange transfusion and any potential harm associated with that procedure. We also assume there was a lack of understanding of the possible side effects, cost, and donor exposure related to IVIG. Additionally, some of those ordering IVIG believe that DAT-negative HDN is common; we have provided additional education to our group, including reference to Dr. Watcko’s recent paper [11] and the new AAP guidelines [12,13], that makes it clear that IVIG is only recommended for DAT-positive HDN. Although not explicitly stated as a criterion in the 2004 AAP guidelines, all of the randomized controlled trials (RCTs) (Table 1) studying the efficacy of IVIG to decrease DVET had enrolled DAT-positive patients. We provided Table 1 locally to our group to demonstrate that all of the randomized controlled trials used DAT-positivity as an entry criteria; thus, the use of IVIG in DAT-negative infants is not “evidence-based.” We used DAT-positivity as a requirement for a diagnosis of “immune-mediated hemolytic disease,” which is consistent with the revised 2022 guideline [12]. The recent publication by Watchko stresses the importance of a positive DAT as part of the diagnosis of ABO hemolytic disease, noting that severe hyperbilirubinemia in an ABO-incompatible neonate with a negative DAT deserves an extensive workup for an alternate cause [11] and should not be attributed to ABO incompatability. After our medication usage analysis, to make our practice “evidence-based,” we developed an institutional guideline that recommended that patients should be DAT-positive. We also recommended that at least a four-hour period of intensive phototherapy is needed prior to ordering IVIG if the TSB is not already within 2–3 of DVET level.

Shortly after manuscript preparation and implementation of our local institutional guideline, the AAP published an update to the 2004 guideline, which clearly states that only infants with a DAT-positive status should be treated with IVIG [12]. In fact, the two major criterion changes in the new 2022 AAP guideline related to IVIG use are (1) DAT positivity and (2) being within 2 of DVET level. However, the separate “technical report” offers an alternate scenario in which IVIG can be considered: “…clinicians may consider the administration of IVIG to infants with isoimmune hemolytic disease (i.e., positive direct antiglobulin test [DAT]) who have not initially responded to phototherapy with a reduction in total bilirubin only in circumstances when the TSB is rising despite intensive phototherapy or within 2 to 3 mg/dL of the exchange level and there is concern that a timely exchange transfusion will be difficult.” [13]. This allows the practitioner some latitude in utilizing IVIG before the TSB is at the “escalation of care, EOC” level, but overall, both the clinical practice guideline and the technical report suggest general restraint in using IVIG aggressively. These updates to recommendations for use of IVIG are valuable clarifications for the practitioner. Therefore, we had to revise our local institutional guideline once again to incorporate this new information (full protocol provided in the Supplemental Text File).

We note several limitations to our study. This was a single-center study, which has a potential bias in local practices influencing the results. In addition, this was a relatively small sample, especially for the examination of adverse effects. Furthermore, as this was a retrospective study, we were unable to collect data on bilirubin levels prior to admission to our hospital system or determine why a neonatologist chose to give IVIG.

Despite the limitations of this study, we felt we needed to raise awareness both locally and nationally that the use of IVIG should be carefully considered. In particular, its use in DAT-negative babies should be avoided. Our criteria for use in our new local protocol is now explicit:

(1) Gestational age ≥ 35 weeks
(2) DAT positive
(3) Either
  - TSB level is at or above the escalation of care (EOC) threshold (2 mg/dL below exchange transfusion level) OR
  - TSB is rising despite intensive phototherapy of 4 h, within 3 mg/dL of the exchange level and/or there is concern that a timely exchange transfusion will be difficult*.

*This exception is primarily intended for DAT+ infants with an unusual antibody for whom DVET blood procurement may be difficult, or for an infant far from a center that performs DVET commonly

The latter criteria is based on the technical report as discussed above, except that we added the “4 h.” We found the main guideline and technical report to be somewhat non-concordant but spoke directly to several of the authors for clarification.

Since we presented our findings locally, we have already noticed that much more discussion and thoughtfulness is occurring before the use of IVIG. One of our criteria in the local protocol is that the attending neonatologist must approve its use, which we believe is leading to more thoughtful consideration of IVIG use. We also believe that protocols serve many purposes, but one purpose is to be educational; thus, we include literature citations within the local guideline so that our residents, fellows, advanced practice providers, and faculty colleagues can avail themselves of the pertinent literature as they wish. This is also why we included the most important information, reported in Table 1 of this manuscript, in the protocol itself, since many of the original randomized controlled trials were conducted years ago and are not necessarily widely known.

## 5. Conclusions

The clinical practice for the use of IVIG in immune-mediated hemolytic disease of the newborn has come under question due to concerns about efficacy in avoiding exchange transfusion, cost, donor exposure, and possible adverse effects including NEC. The technical report that accompanies the most recent AAP guideline states that there is “limited evidence for effectiveness with some evidence of risk of harm support the revised recommendations to limit IVIG use” [13]. In examining our own usage, we found that we were not always compliant with the 2004 AAP guideline that we intended to guide our practice during the study period. After this study, to encourage more evidence-based practice, we developed a new institutional guideline for the use of IVIG in severe hyperbilirubinemia, which we immediately revised (Supplemental Text File) based on the publication of the 2022 AAP guideline and accompanying technical report. Despite the previous randomized trials and the detailed and voluminous data upon which the AAP guidelines are based, we await future studies designed to determine with greater precision the effectiveness and safety of IVIG in patients with immune-mediated hemolytic disease.

## Figures and Tables

**Table 1:** Studies used in the 2018 Cochrane Review ^†^.

Study (Author Year)	Antibody Status of Subjects	IVIG Dose	IVIG Frequency	Conditions for IVIG Administration
Rubo (1992) ^	Rh disease DAT positive	0.5 g/kg	×1	Initiated as soon as Rh disease established
Dagoglu (1995) ^	Rh disease DAT positive	0.5 g/kg	×1	Initiated immediately after birth (typically within 2 h)
Alpay (1999) ^	Rh and/or ABO disease DAT positive	1 g/kg	×1	Bili > 12 mg/dL + retic > 10% (mean time of initiation was HOL 51.5)
Miqdad (2004)	ABO disease DAT positive	0.5 g/kg	×1	Bili rising ≥0.5 mg/dL per h
Nasseri (2006)	Rh or ABO disease DAT positive	0.5 g/kg	Q12 h × 3 doses	Bili rising ≥ 0.5 mg/dL per h (mean time of initiation was HOL 22.8)
Elafly (2011)	Rh disease DAT positive	0.5 g/kg and 1 g/kg in separate cohorts	×1	Administered at HOL 12 if phototherapy required and/or bili rising ≥ 0.5 mg/dL per h
Smits-Wintjents (2011) *	Rh disease DAT positive	0.75 g/kg	×1	Initiated immediately after birth (within 4 h)
Santos (2013) *	Rh disease DAT positive	0.5 g/kg	×1	Initiated immediately after birth (within 6 h)

^†^ Zwiers_C, Scheffer-Rath_MEA, Lopriore_E, de Haas_M, Liley_HG. Immunoglobulin for alloimmune hemolytic disease in neonates. Cochrane Database of Systematic Reviews 2018, Issue 3. Art. No.: CD003313. DOI: 10.1002/14651858.CD003313.pub2; ^ studies available for 2004 AAP guideline; * placebo-controlled studies; IVIG, intravenous immunoglobulin; DAT, direct antiglobulin test; HOL, hour of life.

**Table 2:** Demographic Data and Baseline Characteristics.

Gestational age, weeks (median, IQR)	39 (37.4, 39.5)
Sex, *n* (%)	
Male	31 (52.5%)
Female	28 (47.5%)
Ethnicity, *n* (%)	
Black/African-American	46 (77.9%)
Multi-racial	2 (3.4%)
White	9 (15.3%)
Unknown	2 (3.4%)
Rh D mismatch, *n* (%)	11 (18.6%)
ABO mismatch, *n* (%)	47 (79.7%)
Positive direct antiglobulin test (DAT), *n* (%)	42 (71.2%)
Hour of life that phototherapy started (median, IQR)	7.35 (5.55, 13.05)
Total duration of phototherapy, hours (median, IQR)	85.9 (65.8, 115)

IQR, interquartile range.

**Table 3:** Median Dose of IVIG Administered.

Dose #	Doses of IVIG Ordered, *n* (%)	Dose (g/kg) of IVIG Given (Median, IQR)	Hour of Life IVIG Received (Median, IQR)
1	59 (100%)	1 (0.99, 1.01), *n* = 59	12.62 (10.08, 24.37), *n* = 59
2	14 (23.7%)	1 (0.99, 1.01), *n* = 14	40.38 (27.77, 63.37), *n* = 14
3	2 (2.3%)	1, *n* = 1	79.5 h, *n* = 1

**Table 4:** Bilirubin levels prior to first IVIG order.

Number of Bilirubin Levels Available (In Our Hospital System Data Base)	Number of Patients (*n* = 59)	Mean (SD) Median (IQR) Of Last TSB (mg/dL) Prior to IVIG	Minimum, Maximum TSB (mg/dL)	Rate of Rise (mg/dL per h) between Two TSB Levels Immediately Prior to IVIG (Mean (SD), Median (IQR))
0	4 (7%)	NA	NA	NA
1	24 (41%)	9.0 (0.43)	5.2, 15.6	-
≥2	31 (52%)	10.7 (3.9)	5.2, 20.6	0.43 (0.83) 0.21 (0, 0.44)
Last TSB prior to IVIG	55	10.0 (3.5) 9.4 (7.6, 11.4)	NA	NA

TSB, total serum bilirubin, IVIG, intravenous immunoglobulin, SD, standard deviation, IQR, interquartile range, NA, not applicable. TSBs from outside our hospital system were not included.

## Data Availability

Not applicable.

## Supplementary Materials

The following supporting information can be downloaded at: https://www.mdpi.com/article/10.3390/children10030496/s1; Guideline for the Use of Intravenous Immunoglobulin (IVIG) for Hyperbilirubinemia due to Isoimmune Hemolytic Disease in the Neonatal Intensive Care Unit (NICU).
