# Supplementary material for: Evaluation of Intravenous Immunoglobulin Administration for Hyperbilirubinemia in Newborn Infants with Hemolytic Disease
Source: Children (Basel). 2023 Mar 2;10(3):496. doi: 10.3390/children10030496 (PMC10047662; doi:10.3390/children10030496)
Supplement: Supplementary file 1 [file children-10-00496-s001.zip › children-2207008-supplementary.pdf]

Guideline for the Use of Intravenous Immunoglobulin (IVIG) for Hyperbilirubinemia due to Isoimmune Hemolytic Disease in the Neonatal Intensive Care Unit (NICU)

1/2023

I. Background: The 2022 AAP Clinical Practice Guideline states that IVIG may be given to infants with immune-mediated hemolysis (ie, positive DAT) when their total serum bilirubin (TSB) levels are at or above the escalation of care (EOC) threshold (TSB within 2mg/dL of the baby's exchange transfusion level)[1]. The accompanying Technical Report to the 2022 AAP guideline reviews the body of literature of use of IVIG and summarizes that there is unclear benefit of use of IVIG to prevent exchange transfusion in infants with isoimmune hemolytic disease, with a possible risk of harm due to necrotizing enterocolitis[2]. This document is a guideline to inform clinical practice in the Rainbow NICU, however attending physicians may choose to vary from the suggestions within based on clinical situation and should document reasoning behind their decision-making if using IVIG in circumstances outside of this guideline.

II. Patients should meet **ALL** of the following criteria:
   1) Gestational age ≥ 35 weeks
   2) DAT positive[3]
   3) Either
      a. TSB level is at or above the escalation of care (EOC) threshold (2mg/dL below exchange transfusion level) OR

[1] Kemper AR et al. Clinical practice guideline revision: management of hyperbilirubinemia in the newborn infant 35 or more weeks of gestation. *Pediatrics*. 2022; 150(3): e2022058859
[2] Slaughter JL et al. Techinical report: diagnosis and management of hyperbilirubinemia in the newborn infant 35 or more weeks of gestation. Pediatrics. 2022; 150(3):e2022058865
[3] Absent a positive DAT, diagnosis of ABO hemolytic disease of the newborn is suspect. A negative DAT in a severely hyperbilirubinemic neonate should trigger a search for an alternative cause. Watchko JF. ABO hemolytic disease of the newborn: a need for clarity and consistency in diagnosis. *J Perinatol*. https://doi.org/10.1038/s41372-022-01556-6.
Also note that babies who have received intrauterine transfusions (e.g. in Rh disease) often have a negative DAT at birth since many of their cells are transfused cells.

b. TSB is rising despite intensive phototherapy of 4 hours, within 3 mg/dL of the exchange level and/or there is concern that a timely exchange transfusion will be difficult.[2, 4]

III. Dosing of IVIG

1) Dose is 0.5 grams/kg[5] administered over 2 hours
2) The dose can be repeated one time 12 hours after the first dose if the patient still meets criteria. If the repeat dose is to be given after a DVET, it can be given sooner than 12 hours from the first dose, if the patient meets criteria.
3) IVIG is considered a "blood derivative," not a "blood product." Signed consent is not needed.

IV. Administration of IVIG

1) IVIG may be administered through a PIV, UVC or PICC line[6] over 2 hours.
2) Administer IVIG in a separate infusion line from other medications and maintenance fluids
3) For doses < 60 mL:
   a. Prime 60 inch clear cap-microbore tubing and 0.2 micron filter per NICU protocol for infusing a drip medication.
   b. IVIG is infused via IV infusion pump using the appropriate guardrails.
   c. Administer a normal saline flush to flush medication through tubing (to ensure entire dose is given). Set pump to run 2 mL flush over 15 minutes. Then continue infusing maintenance fluids or saline lock IV.
4) For doses ≥ 60 mL:
   a. Spike bag with IV tubing and standard fluid filter per NICU fluid administration protocol (priming with only medication)
   b. IVIG is infused via IV infusion pump using the appropriate guardrails.
   c. Hang maintenance fluid to flush medication through tubing (to ensure entire dose is given). Set pump to run 20 mL saline flush at the same infusion rate as the IVIG dose and complete with saline flush to IV cap. Then continue maintenance fluids or saline lock IV.
   d. Compatible fluids to flush with include D10W, D10 ¼NS, D5W, D5 ¼NS, or NS.
   e. Do not use fluids containing electrolytes or TPN to flush IVIG.

---

[4] This exception is primarily intended for DAT+ infants with an unusual antibody for whom DVET blood procurement may be difficult, or for an infant far from a center that performs DVET commonly.

[5] Most common dose used in the literature included in the most recent Cochrane Review. Zwiers C et al. Immunoglobulin for alloimmune hemolytic disease in neonates. Cochrane Database of Systematic Reviews 2018, Issue 3. Art. No.: CD003313.DOI: 10.1002/14651858.CD003313.pub2. Literature suggests risk of hemolysis increases with dose. Administration over 2 hours is recommended by the 2022 AAP guideline.

[6] While these are the most optimal sites, a UA line or low-lying UV line could be used but there is no data to support this practice.

V. Monitoring

1) Vital signs are to be monitored prior to starting the infusion, in the middle of the infusion (after 1 hour) and after the infusion is complete
2) If the patient is NPO and maintenance fluids must be stopped for the IVIG infusion, check glucose level in the middle of the infusion (after 1 hour)

VI. Possible adverse events in neonates:

1) Hemolysis
2) Hypoglycemia (most likely related to giving IVIG in single infusion line without maintenance glucose infusing in a second infusion line)
3) Necrotizing enterocolitis in babies ≥34 weeks
4) Donor exposure (1,000 -15,000 donors per dose)
